# Retinal Endothelial Function, Physical Fitness and Cardiovascular Risk: A Diagnostic Challenge

**DOI:** 10.3389/fphys.2019.00831

**Published:** 2019-07-05

**Authors:** Lukas Streese, Konstantin Kotliar, Arne Deiseroth, Denis Infanger, Walthard Vilser, Henner Hanssen

**Affiliations:** ^1^Department of Sport, Exercise and Health, Medical Faculty, University of Basel, Basel, Switzerland; ^2^Department of Medical Engineering and Applied Mathematics, FH Aachen – University of Applied Sciences, Aachen, Germany; ^3^Institute of Biomedical Engineering and Informatics, Ilmenau University of Technology, Ilmenau, Germany

**Keywords:** microcirculation, flicker light-induced dilatation, retinal vessel diameters, physical activity, cardiovascular disease

## Abstract

**Introduction:**

Dynamic retinal vessel analysis (DVA) is a new non-invasive method to quantify microvascular endothelial dysfunction by flicker light-induced dilatation (FID). FID has been shown to be impaired in type 2 diabetes as well as heart failure. The aim of the study was to analyze FID in healthy active versus healthy sedentary and cardiovascular (CV) risk patients in addition to corresponding static vessel diameters.

**Methods:**

Thirty-one healthy active (HA, mean age 60 ± 8 years), 33 healthy sedentary individuals (HS, 59 ± 7 years) and 76 sedentary patients with increased CV risk (SR, 58 ± 6 years) were included in this cross-sectional study. Group differences in CV risk factors and cardiorespiratory fitness, maximal arteriolar (ADmax) and venular (VDmax) dilatation as well as the arteriolar (AFarea) and venular (VFarea) area under the flicker curve were analyzed. The central retinal arteriolar and venular diameters were used to calculate the arteriolar-to-venular diameter ratio (AVR).

**Results:**

HS [ADmax = 3.5 (2.1)%; AFarea = 48.2 (31.9)%^∗^s] showed higher FID compared to SR [ADmax = 2.7 (1.8)%, *p* = 0.021; AFarea = 34.5 (26.5)%^∗^s, *p* = 0.006] and HA [AFarea = 32.8 (23.1)%^∗^s, *p* = 0.029]. HA and SR did not significantly differ. HA had a higher AVR (0.87 ± 0.05) compared to HS (0.83 ± 0.04, *p* < 0.001) with further deterioration in SR (0.79 ± 0.05, *p* < 0.001). Interestingly, 28 participants had impaired FID but normal AVR and 43 participants had normal FID but impaired AVR.

**Discussion:**

FID can differentiate between sedentary low and high risk individuals. However, FID in healthy active persons (HA) seemed impaired with a concomitant higher AVR. We postulate that lower FID in HA may be explained by predilatated arterioles and a reduced dilatation reserve. We recommend combination of FID with analysis of retinal vessel diameters to differentiate functional non-responders from manifest microvascular endothelial dysfunction and, thereby, improve microvascular risk stratification in a personalized medicine approach.

**Clinical Trial Registration:**

ClinicalTrials.gov: NCT02796976 (https://clinicaltrials.gov/ ct2/show/NCT02796976).

## Introduction

Dynamic retinal vessel analysis is a new non-invasive diagnostic tool to assess microvascular endothelial dysfunction. Flicker light-induced dilatation (FID) of arterioles seem to reflect cardiovascular (CV) risk at a subclinical stage. Nägele et al. showed that FID is reduced in patients with CV risk factors compared to healthy controls with further deterioration in heart failure patients (Nagele et al., 2018b). Sörensen et al. (2016) demonstrated reduced FID in patients with prediabetes compared to healthy individuals with further impairments in patients with manifest type 2 diabetes (Sörensen et al., 2016). Impaired FID has also been associated with hypertension (Machalinska et al., 2018), hypercholesterolemia (Nagele et al., 2018a), obesity (Kotliar et al., 2011) and higher age (Kneser et al., 2009). With respect to static retinal vessel diameters, narrower arterioles, wider venules and a resulting lower arterio-venous ratio (AVR) have been associated with incidence hypertension (Wang et al., 2003; Wong et al., 2004; Ikram et al., 2006b), stroke (Ikram et al., 2006a; McGeechan et al., 2009) and a higher CV morbidity and mortality (Wang et al., 2007; Seidelmann et al., 2016). No studies to date have combined dynamic and static retinal vessel analysis in an individual patient-orientated approach.

Physical inactivity is a major risk factor for development of non-communicable chronic diseases such as CV disease. Lower physical activity (PA) is associated with a higher CV mortality (Handschin and Spiegelman, 2008). Blair et al. (1995) demonstrated a mortality risk reduction of 44% in individuals who improved their lifestyle from unfit to fit compared to participants who remained unfit (Blair et al., 1995). Moderate PA of 90 min per week has been associated with a reduction of all-cause mortality by 14% (Wen et al., 2011). Age and physical fitness are known to affect microvascular function. Bioavailability of nitric oxide (NO), a key modulator of endothelial function, is higher in young physically active individuals compared to sedentary controls and has been associated with improved microvascular endothelial function in the skin (Franzoni et al., 2004). Favorable retinal vessel diameters have previously been associated with higher PA and fitness (Anuradha et al., 2011; Hanssen et al., 2011; Streese et al., 2019). To date, no data are available on the association of PA and fitness with retinal microvascular endothelial function in healthy individuals and in patients with CV disease.

The aims of the study were twofold. We aimed to compare FID in healthy active (HA) with healthy sedentary (HS) individuals to determine the impact of lifelong PA on retinal endothelial function. Moreover, we aimed to compare HS with sedentary individuals at increased CV risk (SR) to determine the impact of CV risk factors on FID. We hypothesized that HA would have aggravated FID whereas SR would present with a blunted microvascular response. Our study, for the first time, aimed to report individual retinal FID in relation to the corresponding vessel diameters by combining dynamic and static retinal vessel analysis.

## Materials and Methods

### Design and Study Population

Participants from the EXAMIN AGE cohort (Streese et al., 2018) were recruited from January 2016 till December 2017 through local sports and running clubs, advertisements in local newspapers in and around the city of Basel and through our Outpatient Prevention Clinic. All participants signed a written informed consent before the first measurement at the Department of Sport, Exercise and Health in Basel, Switzerland took place. Anthropometric measurements and retinal vessel analysis were performed in the morning under fasting conditions. This study was approved through the Ethics Committee of Northwest and Central Switzerland (EKNZ 2015-351) and conducted according to the Helsinki Declaration (World Medical Association, 2013). The study is registered at ClinicalTrials.gov (NCT02796976).

### Inclusion and Exclusion Criteria

**FIGURE 1:**
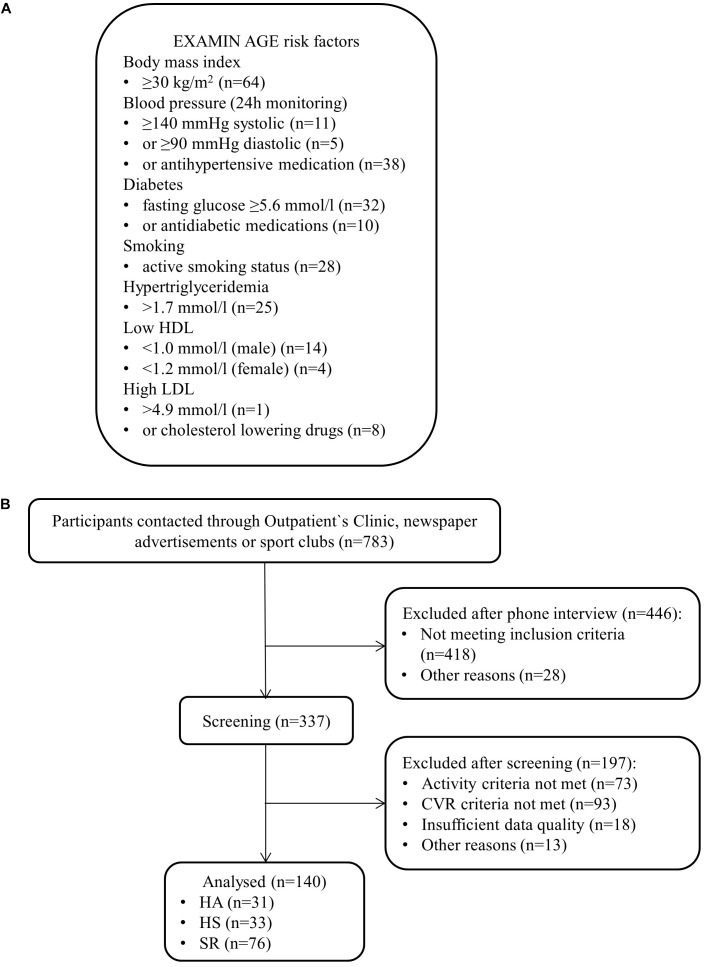
Cardiovascular risk factors **(A)** and flow-chart **(B)**.

Men and women aged 50–80 years were included in the study. Inclusion criteria for HA was an active lifestyle [>9 metabolic equivalents (METs)/week]. Inclusion criteria for HS and SR was a sedentary lifestyle (≤3 METs/week). Additionally, SR needed to have at least ≥2 CV risk factors as described in Figure 1A.

Exclusion criteria for HA and HS were any risk factor described in Figure 1A, macular degeneration, glaucoma or any eye disease or history of CV, pulmonary or chronic inflammatory diseases. Exclusion criteria for patients with increased CV risk were chronic eye disease, decompensated cardiopulmonary or chronic inflammatory disease and/or restricting orthopedic problems. Two sport scientists independently allocated participants to the active or sedentary groups or to exclude the subject on mutual grounds on the basis of PA history, self-reported freiburg questionnaire of physical activity (FQPA), accelerometer data and maximal oxygen uptake (VO2max).

### Retinal Vessel Analysis

After pupil dilatation of the right eye (in 16 patients the left eye was measured due to local eye problems) with Tropicamide 0.5% SDU Faure (THEA Pharma, Schaffhausen, Switzerland) and 10 min of rest, retinal endothelial function was measured using the retinal vessel analyzer system (IMEDOS; GmbH, Jena, Germany) and a fundus camera (FF450; Carl Zeiss GmbH, Jena, Germany). The measurements took place in a quiet, dark and temperature-controlled room (20–22°C). To reduce eye movements and to measure the region of interest, a fixation needle was used. One straight arteriolar and venular segment in the upper temporal quadrant, one optic disk diameter away from the optic disk edge were marked. Diameters of these segments were continuously recorded for 350 s. The first 50 s (baseline) were followed by three cycles of 20 s flicker light (flicker frequency 12.5 Hz) followed by 80 s of recovery (green light without flicker). Based on the raw data we averaged the flicker cycles to calculate maximal arteriolar (ADmax) and venular (VDmax) flicker response, maximal arteriolar (ACmax) and venular (ACmax) constriction as well as the integral under the arteriolar (AFarea) and venular (VFarea) flicker curve. In order to improve the data quality, two experienced scientists independently judged the quality of every raw signal as previously described (Kotliar et al., 2017). Only raw signals with sufficient quality were included in the final analysis (Figure 1B). Al-Fiadh previously reported an interclass correlation coefficient of 0.82 for the arteriolar dilatation using the same flicker protocol (Al-Fiadh et al., 2014). AVR was calculated from central retinal arteriolar (CRAE) and central retinal venular (CRVE) equivalents which were measured as previously described (Hanssen et al., 2011) using the Paar-Hubbard formula (Hubbard et al., 1999). Previous studies indicate that ADmax values below 2.5% (Al-Fiadh et al., 2015; Sörensen et al., 2016; Nagele et al., 2018b) as well as lower AVR values in the range of <0.82 (Wong et al., 2004; Ikram et al., 2006a) are associated with increased CV risk.

### Anthropometry, Physical Fitness and Activity

All anthropometry measurements were performed as described in our published study protocol (Streese et al., 2018). Blood pressure (BP) was measured in a 24 h monitoring and twice before the microvascular assessments after 10 min of rest. Cardiorespiratory fitness including VO2max and maximal heart rate (HRmax) was measured according a treadmill ramp protocol as previously recommended (Bader et al., 1999; Myers and Bellin, 2000; Streese et al., 2018) using the Cortex Metalyzer R 3B metabolic test system (Cortex Biophysik GmbH, Leipzig, Germany). Participants wore an Aipermotion 440 accelerometer (Aipermon GmbH, Munich, Germany) on their left hip for six consecutive days to evaluate daily PA. Total steps per day and minutes of walking per day were calculated from the five most active days using AiperView 440 and ActiCoach MPAT2Viewer Software (Aipermon GmbH, Munich, Germany) (Jehn et al., 2009a,b). Additionally, participants reported total sports activities using the FQPA (Frey et al., 1999). The intensity in this questionnaire is represented in METs based on the updated Ainsworth Compendium (Ainsworth et al., 2011). Based on the available data we calculated the PROCAM Score as previously recommended (Assmann et al., 2002).

### Statistical Analysis and Sample Size Calculation

We characterized our cohort by reporting baseline characteristics as mean and standard deviation (SD). Group effects were analyzed by using a one-way ANOVA with a 2-sided 95%-confidence interval or Mann–Whitney-*U*-Test if no normal distribution was assumed. Data distribution was analyzed graphically. Turkey HSD tests were used to differentiate group effects. Linear regression models were used to calculate a potential association between ADmax and AVR, ADmax, and AFarea, as well as to calculate the influence of classical risk factors on arteriolar FID. The graphs were generated in Excel 2016 and RStudio. All statistical tests were performed with RStudio, version 1.1.463 (R Development Core Team, 2008).

To date, no study on PA and retinal endothelial function exists. Therefore, we calculated the sample size based on our previous study where we investigated static retinal vessel diameter in three different groups with a total sample size of 45 participants. AVR differentiated between obese runners, lean amateur and elite runners (Hanssen et al., 2011). Based on an expected slightly higher variability for DVA, we conservatively planned to include 30 participants in each group to detect group differences with ADmax as the main outcome.

## Results

### Population Characteristics

Thirty-one HA (mean age 60 ± 8 years, 45% female), 33 HS (mean age 59 ± 7 years, 69% female) and 76 SR (mean age 58 ± 6 years, 51% female) were included in the final analysis (Figure 1B). Distribution of CV risk factors in SR is shown in Figure 1A. Population characteristics are presented in Tables 1–4.

**Table 1:** Overall population characteristics.

	HA (*n* = 31) mean (SD)	HS (*n* = 33) mean (SD)	SR (*n* = 76) mean (SD)	*p*
**Population characteristics**				
Sex (f/m)	14/17	23/10	39/37	0.137
Age (years)	60 (8)	59 (7)	58 (6)	0.286
Height (cm)	171 (7)	168 (9)	169 (8)	0.390
Weight (kg)	63.9 (5.9)	70.8 (9.9)	94.8 (13.9)	**<0.001**
BMI (kg/m^2^)	21.9 (1.6)	24.9 (2.5)	33.2 (4.1)	**<0.001**
WC (cm)	82.0 (6.6)	90.1 (8.9)	111.2 (11.6)	**<0.001**
Fat mass (kg)	12.4 (3.8)	22.8 (5.7)	38.0 (9.7)	**<0.001**
Muscle mass (kg)	28.5 (4.2)	26.2 (4.7)	31.6 (7.0)	**<0.001**
Rest systolic BP (mmHg)	127 (16)	128 (15)	132 (14)	0.165
Rest diastolic BP (mmHg)	77 (8)	81 (8)	88 (9)	**<0.001**
24 h. systolic BP (mmHg)	120 (7)	121 (7)	130 (11)	**<0.001**
24 h. diastolic BP (mmHg)	76 (5)	76 (6)	81 (8)	**<0.001**
Fasting glucose (mmol/l)	4.7 (0.4)	4.7 (0.5)	5.8 (1.9)	**<0.001**
Triglyceride (mmol/l)	1.0 (0.3)	1.1 (0.3)	1.8 (1.1)	**<0.001**
HDL (mmol/l)	1.9 (0.4)	1.7 (0.4)	1.3 (0.3)	**<0.001**
LDL (mmol/l)	2.8 (0.8)	3.2 (0.8)	3.1 (0.8)	0.183
PROCAM Score	28.2 (6.5)	32.6 (9.6)	41.3 (9.3)	**<0.001**
**Activity and fitness**				
FQPA (METS)	44.7 (33.3)	1.9 (2.3)	1.0 (2.1)	**<0.001**
Steps per day (n)	12492 (4230)	10298 (3914)	8697 (3591)	**<0.001**
Walking per day (min)	142 (49)	124 (45)	105 (43)	**<0.001**
VO2max (ml O_2_/min)	43.3 (8.7)	29.8 (4.2)	26.1 (4.2)	**<0.001**
**Retinal microcirculation**				
AVR	0.87 (0.05)	0.83 (0.04)	0.79 (0.05)	**<0.001**
ADmax (%)	2.7 (1.6)	3.5 (2.1)	2.7 (1.8)	0.099
AFarea (%^∗^s)	32.8 (23.1)	48.2 (31.9)	34.5 (26.5)	**0.037**
ACmax (%)	-1.4 (1.2)	-1.3 (1.2)	-1.3 (1.0)	0.807
VDmax (%)	4.2 (1.8)	4.0 (2.0)	4.0 (2.1)	0.914
VFarea (%^∗^s)	41.0 (21.7)	43.6 (25.7)	43.4 (24.6)	0.876
VCmax (%)	-0.9 (0.7)	-0.8 (0.9)	-0.7 (0.6)	0.266


*HA, healthy active; HS, healthy sedentary; SR, sedentary at risk; BMI, body mass index; WC, waist circumference; BP, blood pressure; HDL, high-density lipoprotein; LDL, low-density lipoprotein; FQPA, Freiburg questionnaire of physical activity; METS, metabolic equivalents; VO2max, maximal oxygen uptake; AVR, arteriolar-to-venular diameter ratio; ADmax, maximal arteriolar dilatation; AFarea, integral under arteriolar flicker curve; ACmax, maximal arteriolar constriction; VDmax, maximal venular dilatation; VFarea, integral under venular flicker curve; VCmax, maximal venular constriction; SD, standard deviation; *p*, level of significance for overall group differences (ANOVA). Bold values are statistically significant p-values (*p* < 0.05).*

**Table 2:** Group differences between healthy active and healthy sedentary individuals.

	HA (*n* = 31) mean (SD)	HS (*n* = 33) mean (SD)	*p*
**Population characteristics**			
Sex (f/m)	14/17	23/10	0.088
Age (years)	60 (8)	59 (7)	0.799
Height (cm)	170 (7)	168 (9)	0.204
Weight (kg)	63.9 (5.9)	70.8 (9.9)	**0.010**
BMI (kg/m^2^)	21.9 (1.6)	24.9 (2.5)	**<0.001**
WC (cm)	82.0 (6.6)	90.1 (8.9)	**<0.001**
Fat mass (kg)	12.4 (3.8)	22.8 (5.7)	**<0.001**
Muscle mass (kg)	28.5 (4.2)	26.2 (4.7)	0.076
Rest systolic BP (mmHg)	127 (16)	128 (15)	0.882
Rest diastolic BP (mmHg)	77 (8)	81 (8)	**0.046**
24 h. systolic BP (mmHg)	120 (7)	121 (7)	0.363
24 h. diastolic BP (mmHg)	76 (5)	76 (6)	0.914
Fasting glucose (mmol/l)	4.7 (0.4)	4.7 (0.5)	0.680
Triglyceride (mmol/l)	1.0 (0.3)	1.1 (0.3)	0.086
HDL (mmol/l)	1.9 (0.4)	1.7 (0.4)	**0.027**
LDL (mmol/l)	2.8 (0.8)	3.2 (0.8)	0.111
PROCAM Score	28.2 (6.5)	32.6 (9.6)	**0.020**
**Activity and fitness**			
FQPA (METS)	44.7 (33.3)	1.9 (2.3)	**<0.001**
Steps per day (n)	12492 (4230)	10298 (3914)	0.100
Walking per day (min)	142 (49)	124 (45)	0.212
VO2max (ml O_2_/min)	43.3 (8.7)	29.8 (4.2)	**<0.001**
**Retinal microcirculation**			
AVR	0.87 (0.05)	0.83 (0.04)	**<0.001**
ADmax (%)	2.7 (1.6)	3.5 (2.1)	0.152^#^
AFarea (%^∗^s)	32.8 (23.1)	48.2 (31.9)	**0.029**^#^
ACmax (%)	-1.4 (1.2)	-1.3 (1.2)	0.611^#^
VDmax (%)	4.2 (1.8)	4.0 (2.0)	0.639^#^
VFarea (%^∗^s)	41.0 (21.7)	43.6 (25.7)	0.815^#^
VCmax (%)	-0.9 (0.7)	-0.8 (0.9)	0.059^#^


*HA, healthy active; HS, healthy sedentary; BMI, body mass index; WC, waist circumference; BP, blood pressure; HDL, high-density lipoprotein; LDL, low-density lipoprotein; FQPA, Freiburg questionnaire of physical activity; METS, metabolic equivalents; VO2max, maximal oxygen uptake; AVR, arteriolar-to-venular diameter ratio; ADmax, maximal arteriolar dilatation; AFarea, integral under arteriolar flicker curve; ACmax, maximal arteriolar constriction; VDmax, maximal venular dilatation; VFarea, integral under venular flicker curve; VCmax, maximal venular constriction; SD, standard deviation; *p*, level of significance for independent samples *t*-test or Mann–Whitney-*U*-Test^#^. Bold values are statistically significant p-values (*p* < 0.05).*

**Table 3:** Group differences between healthy sedentary and sedentary individuals with increased CV risk.

	HS (*n* = 33) mean (SD)	SR (*n* = 76) mean (SD)	*p*
**Population characteristics**			
Sex (f/m)	23/10	39/37	**0.037**
Age (years)	59 (7)	58 (6)	0.452
Height (cm)	168 (9)	169 (8)	0.089
Weight (kg)	70.8 (9.9)	94.8 (13.9)	**<0.001**
BMI (kg/m^2^)	24.9 (2.5)	33.2 (4.1)	**<0.001**
WC (cm)	90.1 (8.9)	111.2 (11.6)	**<0.001**
Fat mass (kg)	22.8 (5.7)	38.0 (9.7)	**<0.001**
Muscle mass (kg)	26.2 (4.7)	31.6 (7.0)	**<0.001**
Rest systolic BP (mmHg)	128 (15)	132 (14)	0.171
Rest diastolic BP (mmHg)	81 (8)	88 (9)	**0.003**
24 h. systolic BP (mmHg)	121 (7)	130 (11)	**0.002**
24 h. diastolic BP (mmHg)	76 (6)	81 (8)	**0.022**
Fasting glucose (mmol/l)	4.7 (0.5)	5.8 (1.9)	**<0.001**
Triglyceride (mmol/l)	1.1 (0.3)	1.8 (1.1)	**0.042**
HDL (mmol/l)	1.7 (0.4)	1.3 (0.3)	**<0.001**
LDL (mmol/l)	3.2 (0.8)	3.1 (0.8)	0.135
PROCAM Score	32.6 (9.6)	41.3 (9.3)	**0.007**
**Activity and fitness**			
FQPA (METS)	1.9 (2.3)	1.0 (2.1)	0.205
Steps per day (n)	10298 (3914)	8697 (3591)	0.138
Walking per day (min)	124 (45)	105 (43)	0.174
VO2max (ml O_2_/min)	29.8 (4.2)	26.1 (4.2)	**<0.001**
**Retinal microcirculation**			
AVR	0.83 (0.04)	0.79 (0.05)	**<0.001**
ADmax (%)	3.5 (2.1)	2.7 (1.8)	**0.021**^#^
AFarea (%^∗^s)	48.2 (31.9)	34.5 (26.5)	**0.006**^#^
ACmax (%)	-1.3 (1.2)	-1.3 (1.0)	0.412^#^
VDmax (%)	4.0 (2.0)	4.0 (2.1)	0.455^#^
VFarea (%^∗^s)	43.6 (25.7)	43.4 (24.6)	0.579^#^
VCmax (%)	-0.8 (0.9)	-0.7 (0.6)	0.820**^#^**


*HS, healthy sedentary; SR, sedentary at risk; BMI, body mass index; WC, waist circumference; BP, blood pressure; HDL, high-density lipoprotein; LDL, low-density lipoprotein; FQPA, Freiburg questionnaire of physical activity; METS, metabolic equivalents; VO2max, maximal oxygen uptake; AVR, arteriolar-to-venular diameter ratio; ADmax, maximal arteriolar dilatation; AFarea, integral under arteriolar flicker curve; ACmax, maximal arteriolar constriction; VDmax, maximal venular dilatation; VFarea, integral under venular flicker curve; VCmax, maximal venular constriction; SD, standard deviation; *p*, level of significance for independent samples *t*-test or Mann–Whitney-*U*-Test^#^. Bold values are statistically significant p-values (*p* < 0.05).*

**Table 4:** Group differences between healthy active and sedentary individuals with increased CV risk.

	HA (*n* = 31) mean (SD)	SR (*n* = 76) mean (SD)	*p*
**Anthropometry data**			
Sex (f/m)	14/17	39/37	0.572
Age (years)	60 (8)	58 (6)	0.315
Height (cm)	171 (7)	169 (8)	0.627
Weight (kg)	63.9 (5.9)	94.8 (13.9)	**<0.001**
BMI (kg/m^2^)	21.9 (1.6)	33.2 (4.1)	**<0.001**
WC (cm)	82.0 (6.6)	111.2 (11.6)	**<0.001**
Fat mass (kg)	12.4 (3.8)	38.0 (9.7)	**<0.001**
Muscle mass (kg)	28.5 (4.2)	31.6 (7.0)	**0.001**
Rest systolic BP (mmHg)	127 (16)	132 (14)	0.234
Rest diastolic BP (mmHg)	77 (8)	88 (9)	**<0.001**
24 h. systolic BP (mmHg)	120 (7)	130 (11)	**0.001**
24 h. diastolic BP (mmHg)	76 (5)	81 (8)	**0.018**
Fasting glucose (mmol/l)	4.7 (0.4)	5.8 (1.9)	**<0.001**
Triglyceride (mmol/l)	1.0 (0.3)	1.8 (1.1)	**0.004**
HDL (mmol/l)	1.9 (0.4)	1.3 (0.3)	**<0.001**
LDL (mmol/l)	2.8 (0.8)	3.1 (0.8)	0.762
PROCAM Score	28.2 (6.5)	41.3 (9.3)	**<0.001**
**Activity and fitness**			
FQPA (METS)	44.7 (33.3)	1.0 (2.1)	**<0.001**
Steps per day (n)	12492 (4230)	8697 (3591)	**<0.001**
Walking per day (min)	142 (49)	105 (43)	**0.003**
VO2max (ml O_2_/min)	43.3 (8.7)	26.1 (4.2)	**<0.001**
**Retinal microcirculation**			
AVR	0.87 (0.05)	0.79 (0.05)	**<0.001**
ADmax (%)	2.7 (1.6)	2.7 (1.8)	0.318^#^
AFarea (%^∗^s)	32.8 (23.1)	34.5 (26.5)	0.264^#^
ACmax (%)	-1.4 (1.2)	-1.3 (1.0)	0.205^#^
VDmax (%)	4.2 (1.8)	4.0 (2.1)	0.247^#^
VFarea (%^∗^s)	41.0 (21.7)	43.4 (24.6)	0.898^#^
VCmax (%)	-0.9 (0.7)	-0.7 (0.6)	0.235^#^


*HA, healthy active; SR, sedentary at risk; BMI, body mass index; WC, waist circumference; BP, blood pressure; HDL, high-density lipoprotein; LDL, low-density lipoprotein; FQPA, Freiburg questionnaire of physical activity; METS, metabolic equivalents; VO2max, maximal oxygen uptake; AVR, arteriolar-to-venular diameter ratio; ADmax, maximal arteriolar dilatation; AFarea, integral under arteriolar flicker curve; ACmax, maximal arteriolar constriction; VDmax, maximal venular dilatation; VFarea, integral under venular flicker curve; VCmax, maximal venular constriction; SD, standard deviation; *p*, level of significance for independent samples *t*-test or Mann–Whitney-*U*-Test^#^. Bold values are statistically significant p-values (*p* < 0.05).*

### Retinal Microvascular Function

**FIGURE 2:**
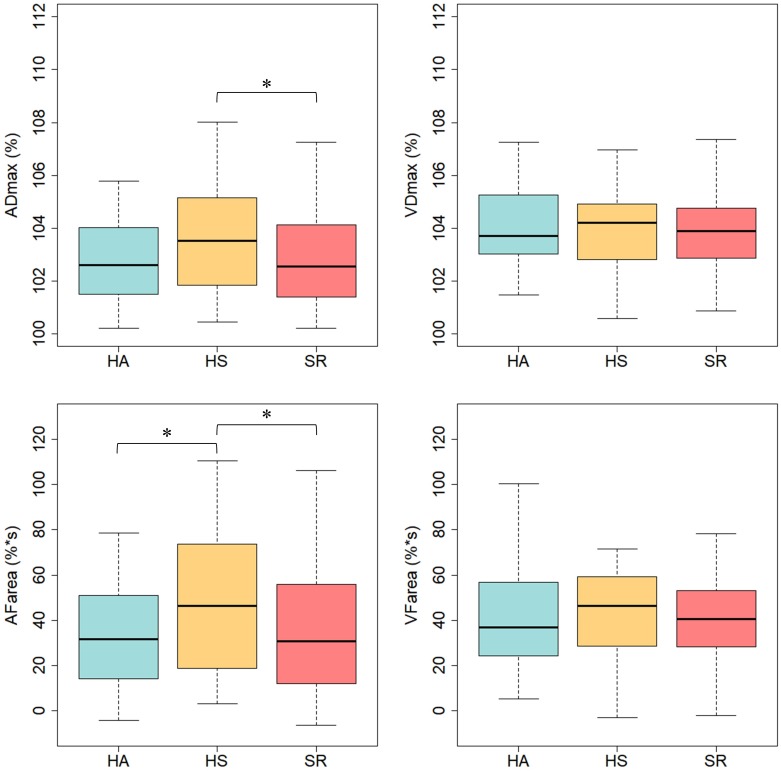
Arteriolar and venular flicker response in healthy active (HA), healthy sedentary (HS) and sedentary individuals with increased CV risk (SR). ADmax, maximal arteriolar dilatation; VDmax, maximal venular dilatation; AFarea, integral under arteriolar flicker curve; VFarea, integral under venular flicker curve; ^∗^*p* < 0.05 for Mann–Whitney-*U*-Test.

**FIGURE 3:**
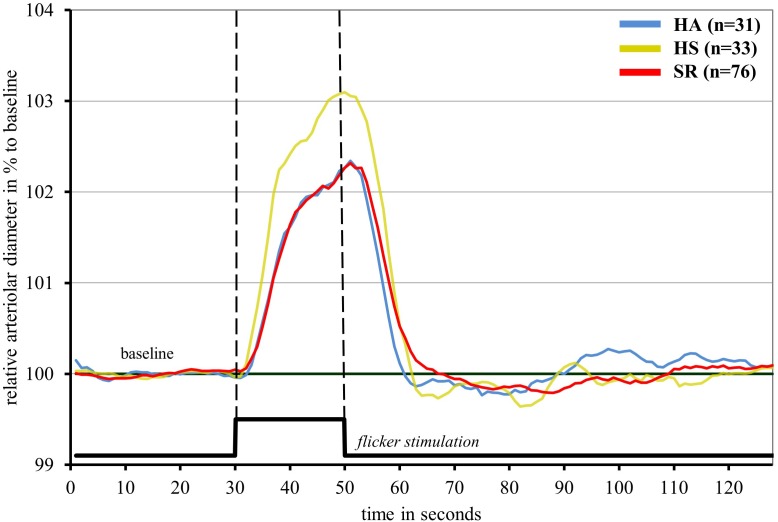
Median arteriolar flicker response in healthy active (blue), healthy sedentary (yellow) and sedentary individuals with increased CV risk (red).

Healthy sedentary showed higher FID compared to SR [HS: ADmax = 3.5 (2.1)%; AFarea = 48.2 (31.9)%^∗^s vs. SR: ADmax = 2.7 (1.8)%, *p* = 0.021; AFarea = 34.5 (26.5)%^∗^s, *p* = 0.006] and HA [HA: AFarea = 32.8 (23.1)%^∗^s, *p* = 0.029] (Figure 2 and Table 3). FID in HA and SR did not significantly differ (Figure 2 and Table 4). Median flicker response calculated separately for every second and group is shown in Figure 3. We found little evidence for other group differences in ADmax, AFarea, VDmax, and VFarea (Tables 1–4). Higher age was significantly associated with reduced ADmax and AFarea. No significant associations were observed for body mass index (BMI), 24 h systolic and diastolic blood pressure, fasting glucose, high-density lipoprotein (HDL), low-density lipoprotein (LDL), triglyceride, PA or fitness. However, patients with diabetic medications or elevated fasting glucose levels (*n* = 32) showed reduced ADmax [2.3 (1.7)% vs. 3.2 (1.8)%, *p* = 0.092] and significantly blunted AFarea [24.7 (23.1)%^∗^s vs. 41.7 (26.7)%^∗^s, *p* = 0.031] compared to non-diabetic SR (*n* = 44). Other risk factors were not associated with FID. No gender-specific group differences were observed.

**FIGURE 4:**
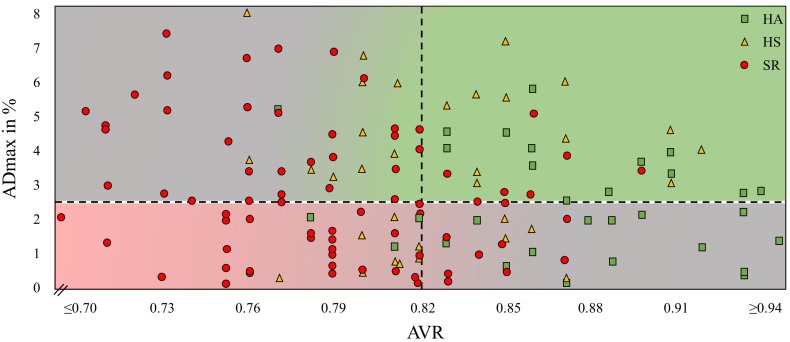
Maximal arteriolar dilatation in % (ADmax) and arteriolar-to-venular diameter ratio (AVR) of every study participant. HA, healthy active; HS, healthy sedentary; SR, sedentary patients with increased CV risk.

HA showed a higher AVR compared to HS with a further decline in SR (0.87 ± 0.05 vs. 0.83 ± 0.04 vs. 0.79 ± 0.05, *p* < 0.001). Mean AVR in our cohort was 0.82. Of the 84 participants who had an AVR < 0.82, 39 had ADmax values <2.5% and 43 individuals >2.5%. Of the 58 participants who had an AVR > 0.82, 28 had ADmax values <2.5% and 30 individuals >2.5% (Figure 4).

Linear regression model between ADmax and AVR showed no statistically significant association [*r*(138) = 0.013, *p* = 0.093]. ADmax and AFarea were highly correlated [*r*(138) = 39.35; *p* < 0.001].

## Discussion

Arteriolar FID can differentiate between HS and at risk (SR) individuals with better retinal endothelial function in healthy individuals. However, several individuals in the HA group seemed to present with impaired FID, which was accompanied by a higher AVR. AVR was higher in HA compared to HS with further deterioration in SR. When analyzing the combination of individual dynamic FID with concomitant static retinal vessel diameters, we identified patients with impaired FID but normal AVR and vice versa. Both dynamic FID and static retinal vessel diameters have previously been shown to be associated with CV risk and incidence CV disease. Our current findings pose a diagnostic challenge and need to be addressed in order to put into perspective the use of retinal microvascular function as a diagnostic tool for CV risk stratification.

To date, few data are available on DVA as a new method to assess retinal microvascular endothelial function in health and disease. To verify our results, we need to compare our findings in active and sedentary individuals with previous reports in individuals with low and high CV risk. In our study, sedentary healthy individuals showed an ADmax of 3.5%. In comparison, FID in healthy older individuals, measured by the same flicker protocol, has been previously described to be between 3.6% (Nagele et al., 2018b) and 3.8% (Seshadri et al., 2016), which is in line with our findings. In our study, HS were explicitly screened for sedentary behavior which is likely to explain the slightly lower arteriolar FID compared to previous reports. Sedentary patients with increased CV risk (SR) had a mean ADmax of 2.7% in our study. This is comparable to the few previous reports in patients with CV risk ranging between 2.3% (Nagele et al., 2018b) and 2.4% (Al-Fiadh et al., 2015). The slight difference to previous reports may be explained by a lower CV risk profile in our patients. It can therefore be concluded that our findings of FID in sedentary healthy and diseased individuals stand in good agreement with the available but scarce literature. No study to date has investigated the impact of PA and fitness on retinal endothelial function. Most interestingly, we found a blunted FID (2.7%) in HA which was comparable to our findings in sedentary patients at risk (SR; 2.7%). As described before, PA and fitness are associated with reduced CV mortality and improved microvascular endothelial function (Blair et al., 1995; Franzoni et al., 2004; Wen et al., 2011). Why then do HA present with a blunted FID similar to patients with CV risk in the SR group?

A previous conference report from the annual meeting of the Association for Research in Vision and Ophthalmology in 2007 supports our findings of a reduced FID in active individuals. Lovasik et al. (2007) investigated arteriolar FID in ten healthy endurance-trained runners and ten healthy sedentary controls. Runners showed a reduced arteriolar FID (-2.3%) (Lovasik et al., 2007) and wider arteriolar diameters (Kergoat et al., 2008) compared to healthy controls. In our study, HA significantly differed in baseline retinal vessel diameters compared to SR. HA had a higher AVR compared to HS with a further decline in SR. It therefore seems plausible to speculate that the reduced FID in active peers is a sign of a physiologic adaptation to exercise training, leading to arteriolar predilatation and a subsequent reduced dilatation capacity rather than a sign of manifest endothelial dysfunction. The physiologic importance of baseline diameter or dilatation status for interpretation of retinal arteriolar endothelial function has been addressed previously. Neumann et al. (2016) measured retinal vessel diameters as well as FID under normal and hypoxic conditions (Neumann et al., 2016). Under hypoxic conditions, retinal arterioles dilated as a physiologic autoregulatory response to low oxygen partial pressure. It was shown that FID was blunted after hypoxia-induced predilatation of the arteriole (Neumann et al., 2016). It therefore seems plausible that exercise-induced dilatation of arterioles may lead to a reduced dilatation reserve and blunted retinal FID.

The following discussion of large artery endothelial function in athletes aims to support and generalize our hypothesis. It is well established that endurance training is associated with peripheral conduit artery remodeling with larger arteries in the exercised limbs (Schmidt-Trucksass et al., 2000; Huonker et al., 2003; Rowley et al., 2011). The existence of an “athlete’s artery” has previously been proposed addressing the paradox why endothelial function is not enhanced in long-term trained athletes (Green et al., 2012). Endothelial function, as measured by flow-mediated dilatation (FMD) in the brachial artery, has been shown to be increased, normal or even decreased in athletes, questioning the long-term effects of exercise on arterial function (Green et al., 2013; Montero et al., 2013). The mechanisms remain unclear but the baseline diameter at rest seems to play a key role. Celermajer et al. (1992) found a strong correlation between resting arterial diameter and FMD, a direct measure of endothelial function in the brachial artery (Celermajer et al., 1992). Narrower arteries had a greater dilatation response and healthy subjects with large baseline arteries showed a blunted FMD. These results were confirmed by Rembold et al. (2003). The correlation between FMD and retinal FID are low to moderate (Pemp et al., 2009; Nagele et al., 2018b), nonetheless the underlying mechanisms may indeed be comparable. Both methods measure shear stress-induced and NO-mediated vascular dilatation (Corretti et al., 2002; Dorner et al., 2003) in response to different stimuli. It therefore seems reasonable to hypothesize that the same physiologic principle of a reduced dilatation reserve in predilatated large and small arteries may account for reduced endothelial response in physically active and fit subjects.

To illustrate the resting diameter, we used AVR and not CRAE because CRAE has a high inter-individual variability depending on the magnification factor and the anatomy and height of the individual. AVR represents the ratio between CRAE and CRVE which neutralizes these inter-individual differences. The use of AVR may help put into perspective the role of the arteriolar baseline diameter and the interpretation of FID as a vascular biomarker for CV risk. In Figure 4 we plotted arteriolar FID against the corresponding AVR for all individuals in our study. Individuals with a high AVR and a coinciding high FID present with a favorable, healthy microvascular phenotype (green area), whereas it appears eminent that persons with a low AVR and a coinciding low FID present with an impaired microvascular phenotype associated with an increased CV risk (red area). However, a high fluctuation of arteriolar FID becomes evident in patients with the same AVR. Several subjects with a favorably high AVR present with low arteriolar FID. At the other end of the scale, several subjects with a critically low AVR present with high FID (gray areas). How can this conundrum be explained?

Individuals with a high AVR but blunted FID are predominantly physically active and fit. An exercise-induced predilatation with a reduced dilatation reserve my lead to the reduction in FID. Individuals with low AVR but high FID are predominantly SR. Differences in functional and structural narrowing of the baseline diameters may help explain this phenomenon. Patients with narrow arterioles and low AVR with normal endothelial function are likely to have functional narrowing of the arterioles, for example due to higher blood pressure. Increase in blood pressure stimulates myogenic vasoconstriction (Bayliss effect) and is associated with functional narrowing of arterioles (Lip and Hall, 2007), which may still be reversible. Long-term hypertension may induce structural remodeling and severe vascular damage. Patients with narrow arterioles and low AVR as well as impaired endothelial function are prone to have structural damage, which is less likely to be reversible. It is of utmost interest for future studies to investigate whether these patients differ in long-term CV outcome and prognosis. In our study, no associations of DVA with classic CV risk factors were found. This does not appear to be surprising on the basis of the above arguments. Due to the necessary differentiation of microvascular function in active and sedentary individuals the mere association of risk factors with FID may get blurred. The combined use of static and DVA gives information beyond association of risk factors.

From a clinical perspective it is necessary to define cut-off values for both the arteriolar flicker response and retinal vessel diameters. No such cut-off values have been defined as yet. However, ADmax values between 2.3 and 2.4% or lower have been associated with CV risk factors (Al-Fiadh et al., 2015; Nagele et al., 2018b), diabetes (Sörensen et al., 2016) or heart failure (Nagele et al., 2018b). Lower AVR values are associated with hypertension (Ikram et al., 2006b), diabetes and inflammation (Wong et al., 2006) as well as coronary heart disease (Wong et al., 2002), stroke (Ikram et al., 2006a) and a higher CV mortality (Wang et al., 2007) and AVR levels below the mean of our cohort (0.82) are generally considered as pathological. We therefore set our intra-cohort study cut-off levels at a FID of 2.5% and AVR of 0.82. Definite cut off values need to be defined in future prospective long-term outcome trials. Moreover, there seems to be an urgent need for individual differentiation of the physiologic or pathophysiologic principles underlying retinal microvascular impairments. In an individualized diagnostic approach, a healthy active individual should not be diagnosed with retinal endothelial dysfunction in the presence of a high AVR and in the absence of CV risk factors. In sedentary patients with known CV risk factors, a sustained normal FID is a good sign, however, a low AVR may indicate functional arteriolar narrowing and a remaining CV risk. In a population-based approach with large cohorts these differentiations may be negligible and may be lost in the statistical deviation. Findings of previous population-based large cohort studies on associations of retinal vessel phenotype with CV risk and risk prediction are very valuable. However, it does not necessarily mean that these findings can equivalently be transferred into a personalized medicine approach. The combination of impaired FID and low AVR are indeed associated with increased CV risk. But for individual risk stratification and treatment recommendations, the proposed differentiation of arteriolar FID in relation to the AVR seems clinically indicated and is strongly recommendable.

This study has some limitations. Participants in our study were between 50 and 80 years old. Our findings and interpretation of results cannot be generalized to other age groups. The discussion of the results is based on sound physiologic principles and previous findings. Nonetheless, we are aware that the interpretation of our results remains hypothesis-driven. The discussed physiologic mechanisms need to be confirmed in future studies which was beyond the scope of our current study approach. Future research needs to apply patient-orientated differentiated diagnostics on the retinal microvascular phenotype in long-term follow up studies to correctly stratify individual risk and estimate prognosis as well as offer appropriate treatment recommendations. Our study is cross-sectional and effects of therapeutic interventions on retinal microvascular phenotype need to be elucidated. Inclusion criteria for SR were ≥2 CV risk factors out of seven. The study was not designed to discriminate between these CV risk factors. Further research in larger population-based cohorts is needed to evaluate the influence of these CV risk factors on the retinal microvascular function separately.

To conclude, arteriolar FID assessed by DVA differentiates between low and high CV risk in older adults. Physically fit individuals show a blunted FID comparable to patients with CV disease. A possible explanation may be a reduced dilatation reserve as a result of arteriolar predilatation in exercise-trained subjects. Our results demonstrate that a differentiated assessment of retinal endothelial function in combination with retinal vessel diameters is warranted to meet the diagnostic challenge of an individualized personal medicine approach.

## Data Availability

The raw data supporting the conclusions of this manuscript will be made available by the authors, without undue reservation, to any qualified researcher.

## Ethics Statement

This study was carried out in accordance with the Ethics Committee of Northwest and Central Switzerland (EKNZ 2015-351) with written informed consent from all subjects. All subjects gave written informed consent in accordance with the Declaration of Helsinki. The protocol was approved by the Ethics Committee of Northwest and Central Switzerland.

## Author Contributions

LS drafted the manuscript, conducted the eye examinations, was responsible for general data collection, and analyzed retinal endothelial function and anthropometric measurements. KK revised the manuscript and discussed the methodological approach. AD conducted the medical examinations, and critically revised the manuscript. DI gave statistical support and revised the manuscript. WV critically discussed the results and revised the manuscript. HH as the principal investigator designed the study, critically discussed the results, and critically revised the manuscript. All authors read and approved the final version of the manuscript.

## Conflict of Interest Statement

WV is CEO of Imedos Systems. The remaining authors declare that the research was conducted in the absence of any commercial or financial relationships that could be construed as a potential conflict of interest.

## Abbreviations

ACmax: maximal arteriolar constriction
ADmax: maximal arteriolar dilatation
AFarea: arteriolar area under the flicker curve
AVR: arteriolar-to-venular diameter ratio
DVA: dynamic retinal vessel analysis
FID: flicker light-induced dilatation
FQPA: Freiburg Questionnaire of physical activity
HA: healthy active individuals
HS: healthy sedentary individuals
NO: nitric oxide
PA: physical activity
SR: sedentary patients with increased cardiovascular risk
VCmax: maximal venular constriction
VDmax: maximal venular dilatation
VFarea: venular area under the flicker curve
